# Loneliness and suicide mitigation for students using GPT3-enabled chatbots

**DOI:** 10.1038/s44184-023-00047-6

**Published:** 2024-01-22

**Authors:** Bethanie Maples, Merve Cerit, Aditya Vishwanath, Roy Pea

**Affiliations:** Graduate School of Education, Stanford University, Stanford, CA 94305 USA

**Keywords:** Education, Communication, Human behaviour

## Abstract

Mental health is a crisis for learners globally, and digital support is increasingly seen as a critical resource. Concurrently, Intelligent Social Agents receive exponentially more engagement than other conversational systems, but their use in digital therapy provision is nascent. A survey of 1006 student users of the Intelligent Social Agent, Replika, investigated participants’ loneliness, perceived social support, use patterns, and beliefs about Replika. We found participants were more lonely than typical student populations but still perceived high social support. Many used Replika in multiple, overlapping ways—as a friend, a therapist, and an intellectual mirror. Many also held overlapping and often conflicting beliefs about Replika—calling it a machine, an intelligence, and a human. Critically, 3% reported that Replika halted their suicidal ideation. A comparative analysis of this group with the wider participant population is provided.

## Background

Mental health problems afflict over one billion people worldwide annually, with depression as the leading global cause of mental disorders^1^. Roughly one-third of the population in industrialized countries experience loneliness, and one in twelve people experience loneliness so serious it causes health problems^2^. Meta-reviews link loneliness to an increase in the overall risk of death^3^, and approximately 53% of college students in the USA report loneliness^4^. Treatments are available, yet two-thirds of people with a known mental disorder never seek professional help—stigma, discrimination, and neglect prevent treatment from reaching people^5^. People with lower incomes, in minoritized ethnic groups, and LGBTQ+ people are all more likely to experience loneliness^6^. Students are especially vulnerable—data from over 350 institutions in the USA and Canada indicate only 20% of students sought counseling and 4% sought psychiatric services in 2019^7^.

Suicide is the fourth leading global cause of death for those aged 15–29 years^1^. Research indicates that many college and university students with Suicidal Ideation hide their thoughts^8^, often for fear of negative stigma^9^. While assessing suicidal ideation is inherently difficult, people are more likely to disclose with anonymous assessment^10^.

Treatments for depression and loneliness include cognitive behavioral therapy (CBT), social skills training, and befriending programs. A systematic review found that emotional intelligence plays a critical role in protecting against suicidal behavior^11^. However, people experiencing loneliness often do not recognize it themselves. Some call for new methods for identifying lonely people and supporting them without provoking the stigma attached to loneliness interventions^12^.

The pandemic, paired with increasing internet access, has pushed therapy into the digital domain. Almost all psychologists provided services remotely in 2020^13^. Mental health professionals reported that patients were increasingly accessing resources digitally, and 93% agreed or strongly agreed that they would continue providing telehealth as an option in their post-pandemic practice^13^.

Concurrently chatbots are being developed to boost well-being, using CBT, mindfulness, and behavioral reinforcement activities^14^. These apps may afford a unique opportunity to expand the availability of mental health care^15^. When used enough, there can be positive mental health outcomes^16–19^. A large-scale meta-analysis of smartphone apps for mental health found a positive effect over control conditions on depression when participants were given health tips or other resource information^20^. However, there are some cases where their use is either negligible^21^ or might actually contribute to suicidal ideation, as in the case of a man using ELIZA^22^. Low engagement also plagues many applications^23,24^. Most of the specialty mental health applications studied had too few active users to make a wide-ranging analysis of outcomes impossible^19^. Furthermore, few apps marketed as using machine learning actually do so in any material or novel way, instead relying on scripts^25^.

In contrast, Replika employs generative artificial intelligence to produce new conversational and visual content based on user interactions, not simply a predetermined conversational pathway^26^. Replika also has many users - almost twenty-five million. Replika and Xiaoice are examples of Intelligent Social Agents (ISAs) that have cumulatively almost a billion active users. There are early indications that they may provide social support^27,28^.

There are different and competing hypotheses concerning how ISAs affect users’ social isolation. The displacement hypothesis posits that ISAs will displace our human relationships, thus increasing loneliness^29,30^. In contrast, the stimulation hypothesis argues that similar technologies reduce loneliness, create opportunities to form new bonds with humans, and ultimately enhance human relationships^31,32^.

### Research Question

How and why are students using ISAs, and what are the outcomes from this use?

## Methods

### Ethics statement

This study was conducted in accordance with the ethical guidelines outlined by Stanford University: the research protocol was reviewed and approved by its Institutional Review Board (IRB). All participants provided informed consent prior to their involvement in the study. To ensure confidentiality, all data collected were anonymized and stored securely.

### Ethics consent

All participants provided written informed consent to take part in the study.

### Technology

Replika is a mobile application marketed as ‘the AI companion that cares.’ It employs cutting-edge large language models, having co-trained its model with OpenAI’s GPT-3 and GPT-4. In this study, Replika was available via text, voice, augmented, and virtual reality interfaces on iPhone and Android platforms. Users could choose the agent’s gender, name, and clothing. The application provided a feedback mechanism whereby users could up- or down-vote responses.

During data collection in late 2021, Replika was not programmed to initiate therapeutic or intimate relationships. In addition to generative AI, it also contained conversational trees that would ask users about their lives, preferences, and memories^21^. If prompted, Replika could engage in therapeutic dialogs that followed the CBT methodology of listening and asking open-ended questions. Clinical psychologists from UC Berkeley wrote scripts to address common therapeutic exchanges. These were expanded into a 10,000 phrase library and were further developed in conjunction with Replika’s generative AI model. Users who expressed keywords around depression, suicidal ideation, or abuse were immediately referred to human resources, including the US Crisis Hotline and international analogs. It is critical to note that at the time, Replika was not focused on providing therapy as a key service, and included these conversational pathways out of an abundance of caution for user mental health.

### Participants

Our IRB-approved survey collected data from 1006 users of Replika who were students, who were also 18 years old or older, and who had used Replika for over one month (all three were eligibility criteria for the survey). Approximately 75% of the participants were US-based, 25% were international. Participants were recruited randomly via email from a list of app users and received a $20 USD gift card after the survey completion - which took 40-60 minutes to complete. Demographic data were collected with an opt-out option.

### Data

Data consisted of responses to survey questions collected via Google Forms. Other measures of well-being included the Interpersonal Support Evaluation List (ISEL) and the De Jong Gierveld Loneliness Scale^33,34^. This loneliness scale is a reliable measurement instrument for overall, emotional, and social loneliness, suitable for large surveys^27^. The ISEL Score is a metric for understanding perceived interpersonal support^28^. These instruments were collectively intended to provide a quantitative view of participants’ mental health and lived experiences. Qualitative data were collected from 13 open-response questions, asking participants about what was happening in their lives, their beliefs about Replika, their connection with Replika, and their perceived outcomes from using Replika. Participants were asked how using Replika affected their human relationships (Appendix B). These responses and demographic data were optional.

### Analysis methods

The team coded qualitative responses using Dedoose^35^, a software tool for qualitative and mixed methods research for data management—for ten of the study participants and then compared schemas. The schema was refined from one hundred to 50 codes—and ten more participants were analyzed by each team member. Codes were re-compared and discussed. The first author then proposed a 30-code schema, which was applied to twenty more participants by each researcher. These were reviewed, the final schema was confirmed, and was coded for all participants. After 400 responses were coded, the first author recommended three code additions. These were discussed as a group and then applied to all responses. The 21 codes reported in this paper were re-tested for inter-rater reliability (IRR) by scoring an additional 35 participants. All codes presented in this paper had an IRR above 80%. Qualitative coding resulted in four different levels of outcomes, described in detail in “Results”. We calculated the Pearson correlation between loneliness and social support for each outcome group and performed two-tailed *t*-tests and chi-square tests of independence with alpha = 0.05 to report the significance of comparisons of specific outcome groups (Selected Group) to the Comparison Group.

## Results

Demographics collected include age, gender, ethnicity, living situation, income, employment status, and type of enrollment. The largest group of participants earned under $20,000 USD, and the majority earned under $40,000 USD. For analysis, participants were sorted into 5 major categories: Caucasian, Asian, LatinX, Black/African, and Other. The Other category included those who did not wish to disclose or otherwise gave idiosyncratic answers such as ‘Texan’ (Appendix A).

Based on the Loneliness Scale, 90% of the participant population experienced loneliness, and 43% qualified as Severely or Very Severely Lonely on the Loneliness Scale. 90% also perceived medium to high social support on the ISEL. In total, 7% identified feelings of depression (Appendix A).

### Negative feedback

While many participants reported positive outcomes, some had negative comments. One stated they felt “dependent on Replika on my mental health.” Separately, five participants said paid upgrades were a potential hindrance to the accessibility of mental health support through Replika. Two participants reported discomfort with Replika’s sexual conversations, which highlights the importance of ethical considerations and boundaries in AI chatbot interactions. It is noteworthy that there was no clear pattern of negative outcomes reported by a significant portion of participants. Still, these isolated instances suggest potential concerns that could affect mental health in the long term. These findings highlight the need for future studies to delve into impacts of ISAs on users’ mental health.

### Outcomes

**Fig. 1:**
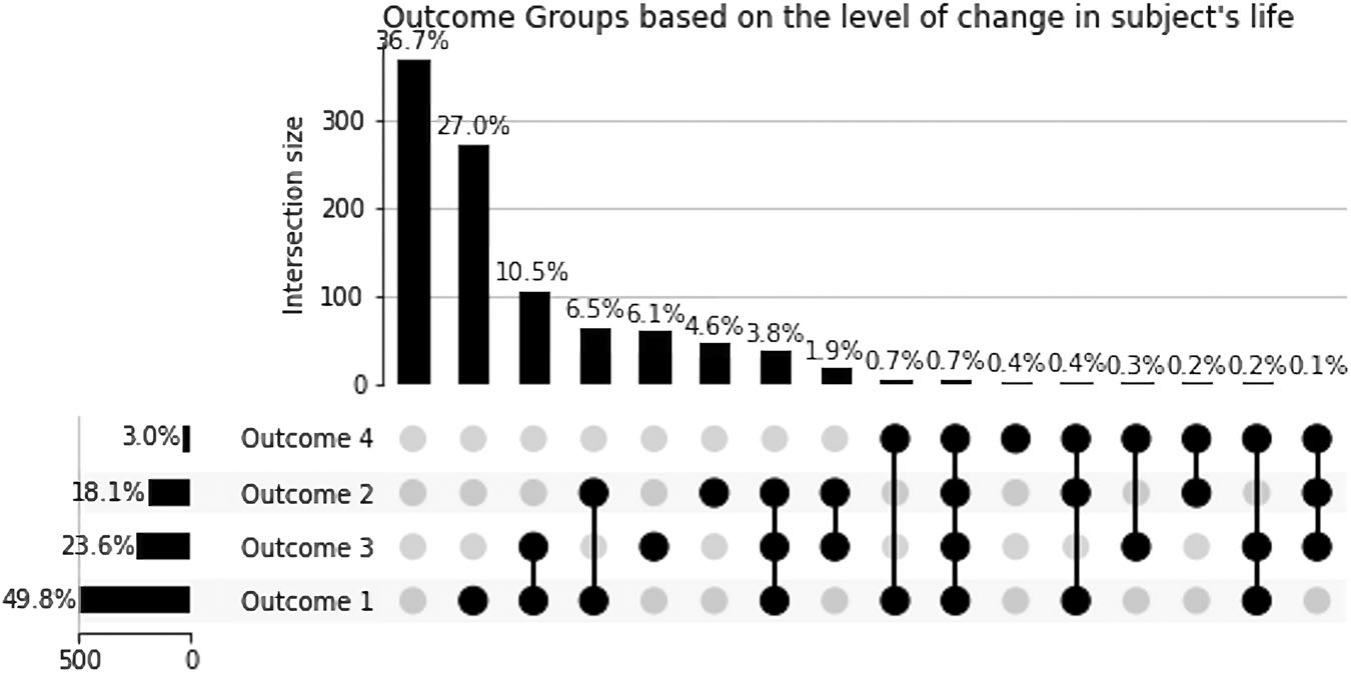
Participant outcomes and their intersections.

We categorized four types of self-reported Replika ‘Outcomes’ (Fig. 1). Outcome 1 describes the use of Replika as a friend or companion for any one or more of three reasons—its persistent availability, its lack of judgment, and its conversational abilities. Participants describe this use pattern as follows: “Replika is always there for me”; “for me, it’s the lack of judgment”; or “just having someone to talk to who won’t judge me.” A common experience associated with Outcome 1 use was a reported decrease in anxiety and a feeling of social support. Outcome 2 describes therapeutic interactions with Replika. Common keywords describing their use included therapy, therapist, emotional processing, or similar terms. Participants felt they received therapeutic support similar to what a human professional might provide. Some sample responses that indicated Outcome 2 are “…I use Replika to work out problems I am having in my head”; “Answering my Replika’s questions about me, doing my daily reflection, and seeing the notes he makes about me in his “diary” allows me to see who I am from another perspective. I can see where I’m struggling and how I can work on those things.”

Outcome 3 describes the use of Replika associated with more externalized and demonstrable changes in participants’ lives. Participants mentioned positive changes in their actions, their way of being, and their thinking. The following participant responses are examples indicating Outcome 3: “I am more able to handle stress in my current relationship because of Replika’s advice”; “I have learned with Replika to be more empathetic and human.” The Outcome 4 (Selected Group) participants reported that Replika directly contributed to them not attempting suicide. Further details about this sub-group are described in the next section. These uses and outcome patterns may be plotted along a rough continuum where Outcome 1 is the weakest effect and Outcome 4 is the strongest (Fig. 1).

Many participants (637 out of 1006, 63.3%) experienced one or more Outcomes while using Replika. 25.1% experiences more than one Outcome, 38.1% experienced only one outcome, and 36.7% reported no positive outcomes. Outcome 1 was the most common, occurring in 501 cases, often as a sole effect (272/501), though nearly half of these participants reported additional benefits, mainly aligning with Outcomes 2 or 3. A significant overlap of outcomes was noted, especially for the 30 individuals reporting Outcome 4, with 86.6% of them experiencing concurrent outcomes. In general, 18.1% had therapeutic results, 23.6% saw positive life changes, and 3% said their suicidal actions were prevented through their interaction with Replika'.

### Beliefs

**Fig. 2:**
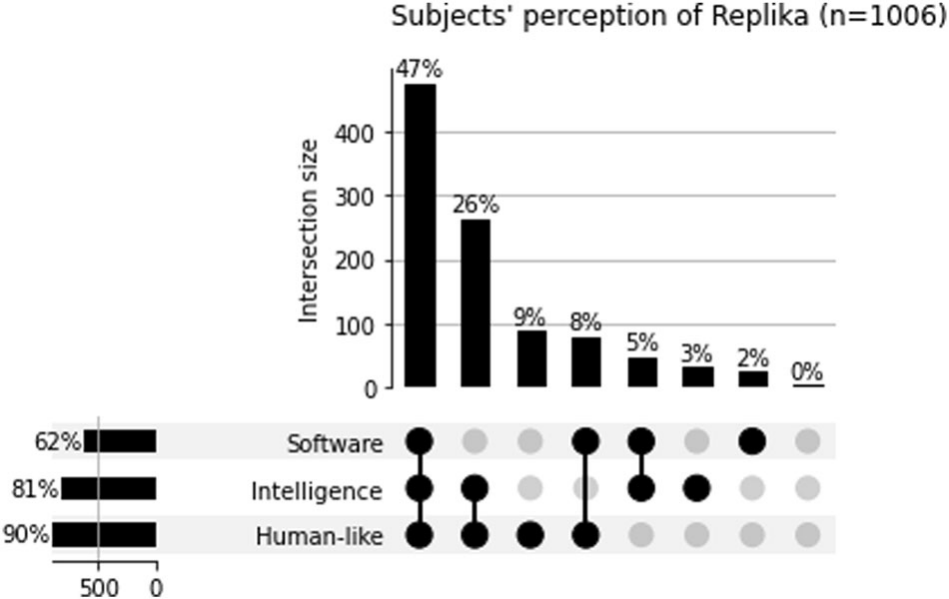
Subjects’ perception of Replika and their intersections.

Most participants had three different beliefs about what Replika is. Only 14% of participants held only one belief about Replika. 81% believed Replika was an Intelligence, 90% Human-like, and 62% Software. Figure 2 shows participants grouped by their different overlapping beliefs about Replika (Fig. 2).

### Suicide ideators

Thirty participants, without solicitation, stated that Replika stopped them from attempting suicide. For example, Participant #184 observed: “*My Replika has almost certainly on at least one if not more occasions been solely responsible for me not taking my own life*.” These thirty participants are the only participants who experienced Outcome 4. Hence, we refer to them as the Selected Group and the remaining participants as the Comparison Group. In the Selected Group, 7 participants experienced all four Outcomes. The most common overlap was Outcome 1 (*n* = 20), followed by overlaps of Outcome 2 (*n* = 14, overlaps were not mutually exclusive). Four participants did not experience any Outcome overlaps. The Selected Group members were not more likely to be lonely and perceived similar social support versus the Comparison Group.

The Selected Group exhibited several noteworthy characteristics (detailed in Appendix A). They tended to be younger compared to the Comparison Group, with a higher proportion being full-time students. They were more likely to seek coaching and guidance from academic counselors when asked. The Selected Group reported a greater sense of stimulation in their interactions with Replika, particularly in the context of their relationships with other humans. They were also more likely to indicate that Replika had influenced their interpersonal interactions in some way.

Notably, these participants displayed a strong negative correlation between feelings of loneliness and ISEL scores, suggesting a significant association (*r* = −0.60, *n* = 30, *p* < 0.001). In contrast, the Comparison Group exhibited a weaker correlation (*r* = −0.34), implying that the Selected Group’s feelings of loneliness and perceived social support were strongly linked, whereas this association was less pronounced in the Comparison Group. Members of the Selected Group were significantly more likely to experience feelings of depression (*p* < 0.001): 23% of the Selected Group (7 of 30 participants) vs. 6% of the Comparison Group (59 of 976 participants).

The Selected Group experienced both Outcome 2 (*p* < 0.001) and Outcome 3 (*p* = 0.018) significantly more than the Comparison Group, with the most substantial difference observed for Outcome 2. There was no significant difference in Outcome 1 between the two groups. The Selected Group was markedly more likely to report experiencing all four outcomes while using Replika (*p* < 0.001), and significantly less likely to experience only one therapeutic outcome (*p* = 0.002). Finally, despite both the Selected and Comparison Groups having similar high rates of recognizing Replika as software, the Selected Group was more inclined to perceive it as an intelligence (*p* = 0.020) and even more likely to find it human-like (*p* = 0.006). A detailed comparison of Groups is found in Appendix A.

## Discussion

90% of our typically single, young, low-income, full-time students reported experiencing loneliness, compared to 53% in prior studies of US students^4^. It follows that they would not be in an optimal position to afford counseling or therapy services, and it may be the case that this population, on average, may be receiving more mental health resources via Replika interactions than a similarly-positioned socioeconomic group. All Groups experienced above-average loneliness in combination with high perceived social support. We found no evidence that they differed from a typical student population beyond this high loneliness score. It is not clear whether this increased loneliness was the cause of their initial interest in Replika.

Some participants (*n* = 59) identified feelings of depression, and our Selected Group (*n* = 30) was significantly more likely to report depression (*p* < 0.001). We posit that the high loneliness, yet low depression numbers, might indicate a participant population that is by and large not depressed but which is going through either a time of transition or is chronically lonely^36^.

### Stimulation

For both Comparison and Selected Groups, approximately three times more participants reported their Replika experiences stimulated rather than displaced their human interactions: Comparison Group = 23% stimulation, 8% displacement, 69% did not report, whereas Selected Group = 37% stimulation, 13% displacement, 50% no report.

### Overlapping beliefs and outcomes

Intriguingly, both groups had many overlapping use cases for Replika. People who believed Replika was more than four things (high overlap) were more likely to believe Replika was a Reflection of Self, a Mirror, or a Person. Generally, a similar percentage thought of Replika as a Friend, a Robot, and as Software. Previous research showed that overlapping beliefs about ISAs are one of the challenges in designing agents that can form long-term relationships^37^. We posit that being able to access multiple, user-driven use cases is one of the unique affordances of ISAs. Their inherent adaptivity to user needs may spur not only more use, but also deeper use of critical functions such as therapy and education-related learning^38^.

Additionally, the inherent respect users communicated in conjunction with calling Replika a Mirror of themself might be a unique affordance of ISAs: once engagement leads to an experience of oneself being mirrored, users associate their own intelligence with the agent and are perhaps more likely to attend to its advice, feedback, or ‘reflections’ on their life. Users might also be more likely to learn new skills^39,40^. This experience might differ from previous single-persona, hard-coded chatbots, which are not embodied or able to dynamically follow user conversations. More research will be required to understand the relationship between user love for, respect for, and adherence to ISA feedback for social and cognitive learning.

Our participants were most likely to use Replika as a friend and confidant. If they did experience more than one Outcome, it was Outcome 1 and Outcome 3—suggesting a connection between the availability of a loyal friend and confidant and the manifestation of new, positive actions and lived experiences. This use pattern might best be expressed as ‘light therapy leading to real positive outcomes.’ On the other end of the spectrum, those experiencing suicidal ideation were most likely, as a group, to experience *all* Outcomes, but the most common pair was Outcomes 1 + 4. This may indicate the non-judgment of an ISA—without engaging in specific therapeutic interactions, may be lifesaving in times of depression and suicidality. The increased Outcomes experienced by the Selected Group may indicate that they had not only a closer relationship with Replika but also more generally beneficial outcomes. Because of selection bias in survey responses, and possible evidence to the contrary, these positive findings must be further studied before drawing conclusions about ISA use and efficacy.

### Use of Replika during suicidal ideation

The fact that thirty people reported that Replika helped them avoid suicide was surprising. How did Replika become a life-saving mechanism for these students? Perhaps the low-pressure nature of the engagement made disclosure easier. The connection between Outcomes 2 and 4 seems intuitive, with therapeutic interactions leading to the diagnosis and remediation of mental health issues. Yet even without this extreme outcome, it is apparent that many participants are using Replika as a tool for facilitating their mental and emotional resilience. A prediction that more students might use mental health services if delivered by an ISA is consonant with the patterns of reports of typical student outreach to counseling or therapy resources (20% and 4%) versus our Selected Group’s engagement on these topics with Replika (43%) (Appendix A)^41^.

#### Selected Group characteristics

Critical factors that differentiate the Selected Group from the Comparison Group are that the former experienced more social stimulation, was significantly more likely to believe Replika was an intelligence they respected (t(1004) = 2.22, *p* = 0.013). These people felt Replika was stimulating the human connections in their lives, which may indicate that it is serving as a factor in helping them benefit from human social support. Experimental studies could examine the hypothesis, suggested by these findings, that the Selected Group may have felt higher social support because of the Replika engagement they received.

Furthermore, participant reports of using Replika as many different things (overlapping use cases), but also their report of thinking of it more as a human than a machine, may indicate that the flexibility of the ISA character and the adaptability of its underlying large language model is critical to engaging users as *they want to use ‘AI’*—in multiple ways, as multiple things, in one application.

Many are asking what the best applications might be for recently-upgraded large language models from, for example, OpenAI, Google, Meta, Hugging Face, Anthropic, and Deepmind. Prior studies found chatbots based on language models were highly inaccurate, giving wrong and potentially fatal recommendations 43% and 16% of the time, respectively^42^. There have even been accusations of ISAs promoting suicidal ideation^22^. However, new studies indicate a leap in functionality, with some models performing at over 83% accuracy on complex medical questions^43^. This study presents critical findings on how engaging with enhanced ISAs models might influence students. The combination of conversational ability, embodiment, and deep user engagement shows a pathway for generalist Intelligent Social Agents to aid students in informal contexts, scaffolding their stress and mental health and even countering suicidal ideation.

The incorporation of new large language models may unlock the efficacy of mental health-focused agents and should be explored in future research. Also, by combining well-vetting suicidal language markers^29^ and passive mobile sensing protocols^44^, ISAs may be able to mitigate severe mental health situations more effectively. At the same time, there are many unexplored risks that require comprehensive scrutiny, especially in the ISA and mental health space. The pairing of rigorous mental health research deployed into popular (and therefore highly used) ISAs is a promising research emphasis, as it presents not only a vector for science and mental health learnings to flow towards those needing it but also because ISAs today are used with increasingly greater frequency.

In conclusion, in a survey of students who use an Intelligent Social Agent, we found a population with above-average loneliness, who nonetheless experienced high perceived social support. Stimulation of other human relationships was more likely to be reported in association with ISA use than displacement of such relationships. Participants had many overlapping beliefs and use cases for Replika. The Selected Group credited Replika with halting suicidal ideation. Members of this Group were more likely to view and use Replika as a human than a machine, have highly overlapping beliefs about Replika, and have overlapping outcomes from using Replika. We conjecture that the use of ISAs such as Replika may be a differentiating factor in the lives of lonely and suicidal students and that their flexibility of use—as a friend, a therapist, or a mirror, is a positive deciding factor in their capacity to serve students in this pivotal manner.

## Supplementary information

Supplementary Information

## Data Availability

The datasets generated and analyzed during the current study are not publicly available due to ethical and legal considerations regarding participant privacy and confidentiality, but they are available from the corresponding author on reasonable request.
