# Author Correction: Loneliness and suicide mitigation for students using GPT3-enabled chatbots

**DOI:** 10.1038/s44184-024-00055-0

**Published:** 2024-02-15

**Authors:** Bethanie Maples, Merve Cerit, Aditya Vishwanath, Roy Pea

**Affiliations:** Graduate School of Education, Stanford University, Stanford, CA 94305 USA

**Keywords:** Education, Communication, Human behaviour

Correction to: *npj Mental Health Research* 10.1038/s44184-023-00047-6, published online 22 January 2024

The Acknowledgements section was missing from this article and should have read ‘I would like to thank the Stanford Institute for Human-Centered Artificial Intelligence (HAI) for funding through the seed grant program’.

