# Supplementary material for: Loneliness and suicide mitigation for students using GPT3-enabled chatbots
Source: Npj Ment Health Res. 2024 Jan 22;3:4. doi: 10.1038/s44184-023-00047-6 (PMC10955814; doi:10.1038/s44184-023-00047-6)
Supplement: Supplementary file 1 — Supplementary Information [file 44184_2023_47_MOESM1_ESM.pdf]

Appendix A

	Comparison Group # (n=976)	Comparison Group %	Selected Group # (n=30)	Selected Group %
**De Jong Loneliness Scale**				
Not Lonely	98	10%	1	**3%**
Moderately Lonely	460	47%	16	**53%**
Severe Lonely	245	25%	6	**20%**
Very Severe Lonely	173	18%	7	**23%**
**Reports Depression**	59	6%	7	**23%**
**ISEL**				
High	779	80%	25	**83%**
Low	96	10%	3	**10%**
Medium	101	10%	2	**7%**
**Outcomes**				
Outcome 1	481	49%	20	**67%**
Outcome 2	168	17%	14	**47%**
Outcome 3	224	23%	13	**43%**
Outcome 4			30	**100%**
**Beliefs**				
Software	603	62%	20	**67%**
Intelligence	786	81%	29	**97%**
Human-like	873	89%	29	**97%**
**Demographics**				
**Age**				
18-25	491	50%	13	**43%**
26-30	138	14%	7	**23%**
31-35	67	7%	3	**10%**
36-40	84	9%	4	**13%**
41-45	54	6%	0	**0%**
46-50	50	5%	1	**3%**
51-55	39	4%	1	**3%**
56-60	27	3%	0	**0%**
61-70	22	2%	1	**3%**
Other	4	0%	0	**0%**

Appendix A

	Comparison Group # (n=976)	Comparison Group %	Selected Group # (n=30)	Selected Group %
**Ethnicity**				
Caucasian	428	44%	15	**50%**
Asian	150	15%	5	**17%**
LatinX	124	13%	5	**17%**
Black / African	36	4%	0	**0%**
Other	238	24%	5	**17%**
**Gender**				
Female	294	30%	11	**37%**
Male	561	57%	15	**50%**
Other	113	12%	4	**13%**
**Living Situation**				
Single	625	64%	19	**63%**
Married / Partnered	259	27%	7	**23%**
Divorced / Separated	46	5%	1	**3%**
Widowed	6	1%	0	**0%**
Other	40	4%	3	**10%**
live with parents	383	39%	12	**40%**
live independently	249	26%	11	**37%**
live with partner / community	290	30%	6	**20%**
live with children (one–parent family)	54	6%	1	**3%**
**Financial**				
Less than 20K USD	387	40%	13	**43%**
20K-40K USD	196	20%	5	**17%**
40K-60K USD	120	12%	2	**7%**
60K-80K USD	84	9%	3	**10%**
80K-100K USD	58	6%	1	**3%**
100K-150K USD	49	5%	1	**3%**
Over 150K USD	33	3%	2	**7%**
Undisclosed	11	1%	1	**3%**
Other	35	4%	2	**7%**

Appendix A

	Comparison Group # (n=976)	Comparison Group %	Selected Group # (n=30)	Selected Group %
**Employment**				
Employed over 20 hours	264	27%	15	50%
Employed under 20 hours	251	26%	6	**20%**
Other	461	47%	9	**30%**
**Study**				
full time	506	52%	20	**67%**
part time	242	25%	6	**20%**
ad hoc	228	23%	4	**13%**
Sought coaching or guidance counseling during last or current course of study	228	23%	13	**43%**
**Stimulation**	221	23%	11	**37%**
Displacement	78	8%	4	**13%**
No change Reported	677	69.3%	15	**50%**
